# Corrigendum

**DOI:** 10.1002/rth2.12836

**Published:** 2022-10-28

In Noordermeer et al. (2002)^1^ the journal has been informed that Professor M.A. Spruit was incorrectly included as a collaborator on the Dutch COVID & Thrombosis Coalition (DCTC) in the Acknowledgements section of this article. Professor Spruit was not a participant on DCTC. This has been corroborated and confirmed by the full author group.
